# Complete genome sequence of Tacaribe virus

**DOI:** 10.1007/s00705-020-04681-9

**Published:** 2020-05-27

**Authors:** Julia Holzerland, Anne Leske, Lucie Fénéant, Dominique Garcin, Daniel Kolakofsky, Allison Groseth

**Affiliations:** 1 Junior Research Group Arenavirus Biology, Friedrich-Loeffler-Institut, Südufer 10, Greifswald, Insel Riems Germany; 2 Department of Microbiology and Molecular Medicine, University of Geneva Medical School, Geneva, Switzerland

## Abstract

Tacaribe virus (TCRV) is the prototype of the New World arenaviruses (also known as TCRV serocomplex viruses). While TCRV is not itself a human pathogen, many closely related members of this group cause hemorrhagic fever, and thus TCRV has long served as an important BSL2 system for research into diverse areas of arenavirus biology. Due to its widespread use, a coding-complete sequence for both the S and L segments of the bipartite genome has been publically available for almost 30 years. However, more recently, this sequence has been found to contain significant discrepancies compared to other samples of the same original strain (i.e., TRVL-11573). Further, it is incomplete with respect to the genome ends, which contain critical regulatory elements for RNA synthesis. In order to rectify these issues we now present the first complete genome sequence for this important prototype arenavirus. In addition to completing the S segment 5’ end, we identified an apparent error in the L segment 3’ end as well as substantial discrepancies in the S segment intergenic region likely to affect folding. Comparison of this sequence with existing partial sequences confirmed a 12-amino-acid deletion in GP, including putative glycosylation sites, and a 4-amino-acid exchange flanking the exonuclease domain of NP. Accounting for these corrections, the TRVL-11573 strain appears to be nearly identical to that isolated in Florida in 2012. The availability of this information provides a solid basis for future molecular and genetic work on this important prototype arenavirus.

Arenaviruses are small RNA viruses with two ambisense genome segments. The large (L) segment encodes the viral polymerase (L) and the matrix protein (Z), while the small (S) segment encodes the glycoprotein (GP) and the nucleoprotein (NP). The open reading frames (ORFs) are separated by a structured non-coding intergenic region (IGR) that facilitates transcription termination (Fig. 1A) [1–3]. Highly conserved sequences at the genome termini (untranslated regions, UTRs) contain conserved complementary nucleotides that are critical for viral RNA synthesis [4–6].

**Fig. 1:**
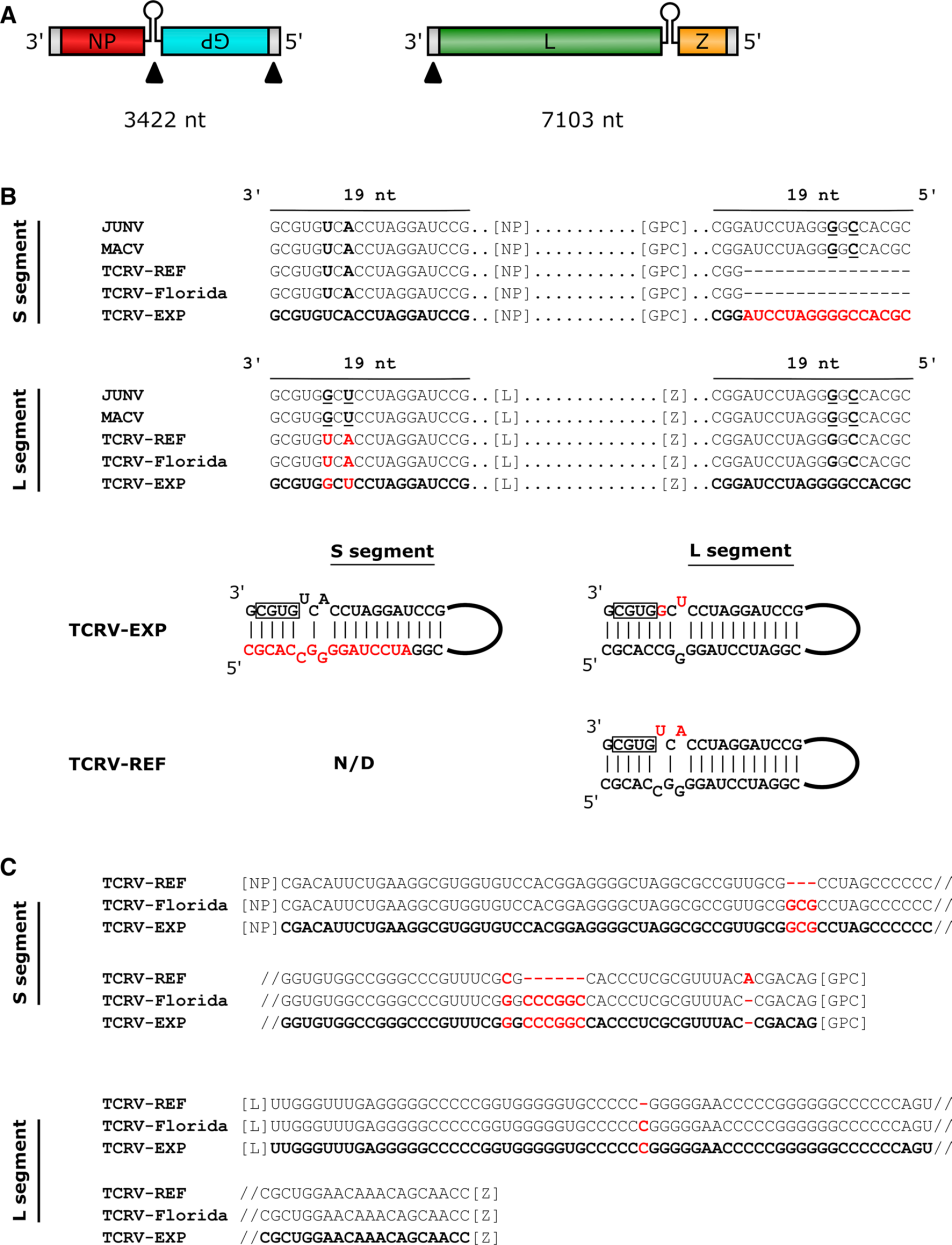
Analysis of Tacaribe virus non-coding sequences. (A) Schematic diagram of the genome, indicating discrepancies in non-coding regions. Open reading frames (colored boxes), terminal noncoding regions (grey boxes) and intergenic regions (hairpin) are all indicated. Arrowheads indicate the location of missing data and discrepancies. **(B)** Genome termini. Sequences determined in this study (TCRV-EXP, shown in bold) were compared to the reference sequences for strain TRVL-11573 (TCRV-REF) and the 2012 Florida strain (TCRV-Florida) as well as Junín virus (JUNV, strain Romero) and Machupo virus (MACV, strain Carvallo). Mismatches are shown in red, and missing data are indicated by dashes. Complementarity of the genome end sequences is shown with the promoter region at the 3’ end boxed. Missing data/discrepancies are shown in red. **(C)** Intergenic regions. Annotation is as described for (B). GenBank accession numbers are as follows: TCRV-EXP (S segment, MT081316; L segment, MT081317), TCRV-REF (S segment, M20304; L segment, J04340), TCRV-Florida (S segment, KF923400; L segment, KF923401). JUNV and MACV S and L segment sequences were as published in references [23] and [24], respectively

The arenaviruses that infect mammals (i.e., mammarenaviruses) are divided into the Old World arenaviruses, which are primarily found in Africa, and the New World arenaviruses, which are mostly found in South America. Tacaribe virus (TCRV; species *Tacaribe mammarenavirus*) is the prototype of such New World arenaviruses (also known as TCRV serocomplex viruses). While many members of this group are causative agents of hemorrhagic fever, TCRV is not itself a human pathogen, making it important both for comparative pathogenesis studies and as a BSL2 system for basic research into arenavirus biology [7, 8].

TCRV was originally isolated from dead bats collected in Trinidad as part of a rabies surveillance program at the Trinidad Regional Virus Laboratory (TRVL). Further efforts led to several additional isolations during the period from 1956 to 58; however, only the strain TRVL-11573 was preserved [9]. It has since been disseminated to laboratories worldwide, where it has formed the basis for all molecular biology research on this virus. Indeed, it remained the only strain in existence until 2012, when a nearly identical virus isolate was recovered from ticks collected in a Florida state park [10]. Unsurprisingly, given its importance for research, sequences for both segments of the TRVL-11573 strain were generated early on and have been available in the GenBank database since 1993 (accession no. M20304 [S], J04340 [L]) [11–14]. The genome sequence established by these reference sequences is coding-complete and has formed an important basis for many molecular and functional studies. However, more recent studies have increasingly suggested that these sequences also contain significant errors [10, 15, 16]. Furthermore, no currently available TCRV sequence includes the 5’ end of the S segment – information that is critical for the development of molecular systems dependent on viral RNA synthesis (e.g., reverse genetics systems). To address these issues, we have generated a complete (end-to-end) genome sequence based on the TCRV prototype strain TRVL-11573 using modern sequencing methods.

## Provenance and sequencing

TCRV (strain TRVL-11573) [9] was obtained through the University of Geneva and was originally sourced from the Arbovirus Reference Laboratory of the CDC [17]. Virus stocks were grown on Vero76 cells (CCLV-RIE0228), and viral RNA was isolated from these supernatants using a QIAamp Viral RNA Mini Kit (QIAGEN) and reverse transcribed using virus-specific primers and Superscript III (Invitrogen). The resulting cDNA was then used with iProof (Bio-Rad) to amplify specific overlapping regions of the genome, which were then purified using a NucleoSpin Gel and PCR Clean-Up Kit (Macherey-Nagel). Genome ends were amplified from cDNA using ligation-anchored PCR, as described previously [18–20]. Briefly, for 3’ end amplification, a 3’-end-blocked linker (/5Phos/GAAGAGAAGGTGGAAATGGCGTTTTGG/3Phos/) was ligated to the viral RNA using T4 RNA ligase (NEB) prior to reverse transcription with a gene-specific primer and subsequent PCR using a gene-specific primer and a primer complementary to the linker sequence. In contrast, for 5’ end amplification, cDNA was synthesized using an internal gene-specific primer and cleaned up using a QIAquick PCR Purification Kit (QIAGEN) prior to linker ligation and PCR as described above. Sanger sequencing of all products with specific primers was performed by Eurofins/GATC. Additional details of the experimental protocols are available on request. IGR folding predictions were performed using Mfold [21].

## Sequence properties

Sequencing of the TCRV genome revealed 7103 nucleotides for the complete L segment (GenBank accession MT081317) and 3422 nucleotides for the complete S segment (GenBank accession MT081316) (Fig. 1A) and identified 16 nucleotides that were missing from the 5’ end of the existing S segment reference sequence (Fig. 1B). Significant discrepancies were also identified in comparison to the previously reported 3’ end sequence of the L segment. Specifically, we observed differences at nucleotides 6 and 8 of the 3’ terminus that change the predicted base pairing between the 5’ and 3’ termini (Fig. 1B). The new sequence data would suggest that the TCRV genome ends are identical to those of the closely related Junín virus (JUNV) and Machupo virus (MACV). Interestingly, it has been reported recently that publically available reference sequences for JUNV and MACV also contained errors at these same positions and that such errors can hamper the development of reverse genetics systems [22–24]. We also identified discrepancies in the IGRs (Fig. 1C). While the single-nucleotide insertion in the L segment IGR appears to have little effect on the energetics of folding, the more extensive changes in the S segment are predicted to have a dramatic effect on the stability of the secondary structures formed in this region (ΔG = -78.0 (vRNA)/76.6 (cRNA) kcal/mol compared to ΔG = -52.4 (vRNA)/53.8 (cRNA) kcal/mol) for the reference sequence). These changes indicate that the IGRs of TRVL-11573 are identical to those reported for the Florida strain (Fig. 1C).

**Fig. 2:**
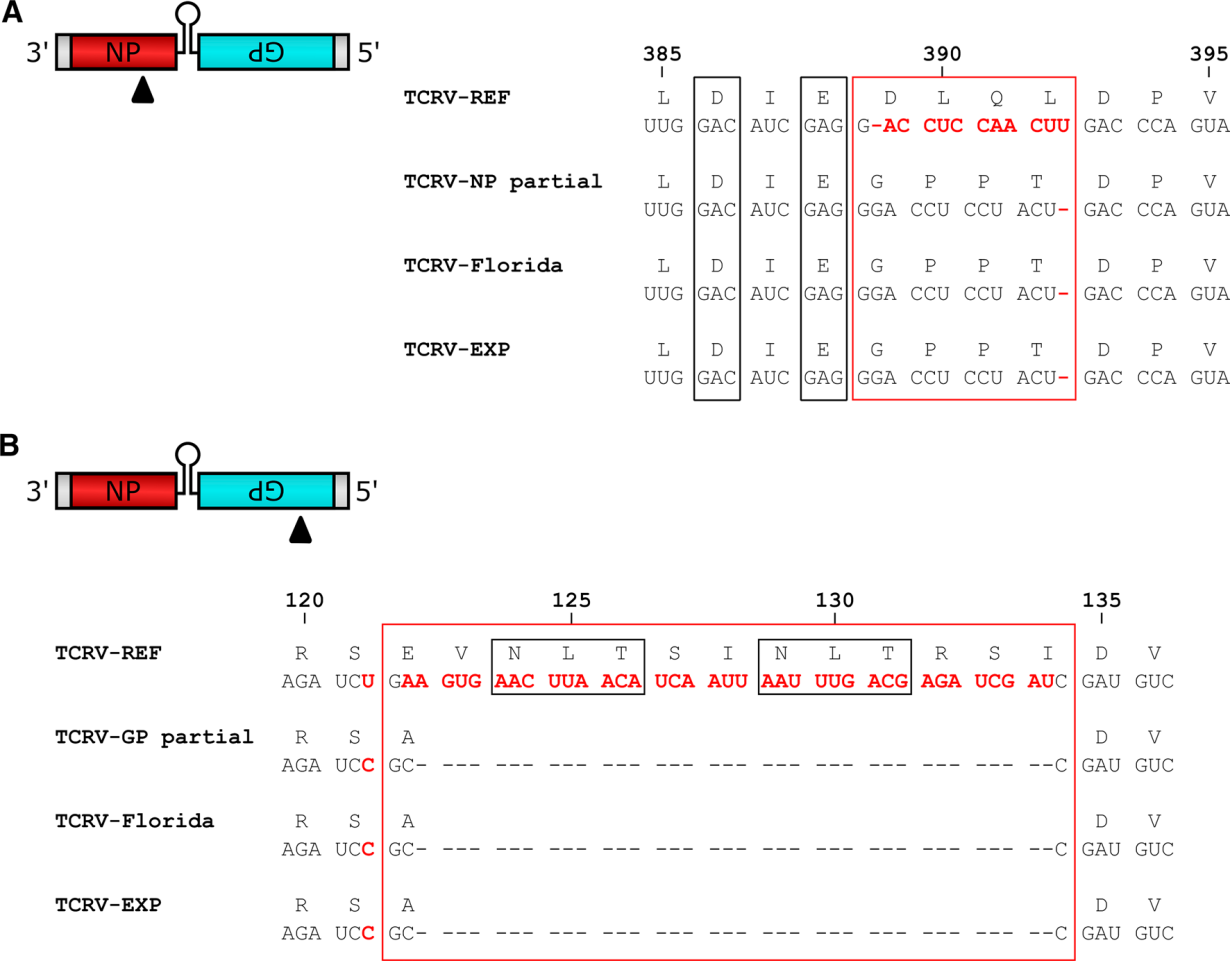
Analysis of Tacaribe virus coding region sequences. Sequence discrepancies in the (A) nucleoprotein (NP) or (B) glycoprotein (GP) open reading frame. Arrowheads indicate the location of discrepancies, which are shown in red text in the respective sequences. Amino acid positions are indicated. Exonuclease catalytic site residues (in NP) and putative N-linked glycosylation sites (in GP) are boxed in black. GenBank accession numbers are as follows: TCRV-EXP (S segment, MT081316; L segment, MT081317), TCRV-REF (S segment, M20304; L segment, J04340), TCRV-Florida (S segment, KF923400; L segment, KF923401), TCRV-NP partial (KC329849), TCRV-GP partial (KP159416)

Comparison of the coding regions also highlighted several obvious differences. In particular, the NP sequence contains two frameshift mutations (a deletion and an insertion) that result in a 4-amino-acid exchange from GPPT to DLQL (Fig. 2A) in a loop region flanking key exonuclease active site residues. While this mutation was originally proposed to explain the reduced ability of TCRV NP, in comparison to other arenavirus NPs, to inhibit type I interferon (IFN) production during infection [25], the presence of a GPPT-to-DLQL mutation could not be confirmed by more-recent sequences derived from the TRVL-11573 isolate [15], nor was it found in the 2012 Florida isolate [10]. Furthermore, the sequence data for GP revealed a 12-amino-acid deletion that eliminates potential N-linked glycosylation sites that are present in the reference sequence. While it is unclear if this difference is due to the loss of this region during virus passaging over the decades, or whether it is due to improvement in sequencing techniques, our observation is consistent with findings from a recently reported partial GP sequence for TRVL-11573 (KP159416) [16] (Fig. 2B) as well as the 2012 Florida strain [10], suggesting that other current isolates also lack this sequence. Overall, the sequences generated in this study support both of these reported deviations from the currently available reference sequence for the TRVL-11573 isolate of TCRV.

Interestingly, taking these discrepancies into account, TRVL-11573 shows a much higher degree of sequence similarity to the sequence isolated from ticks in Florida than was reported based on the previously available reference sequence [10], with the S segments showing 99.7% identity (9 nucleotide mismatches; 6 amino acid changes) and the L segments showing 99.9% identity (10 nucleotide mismatches; 2 amino acid changes). Indeed, when all publically available TRVL-11573 sequences, including partial sequences, are taken into account, only one nucleotide position in the S segment and two in the L segment appear to be unique to the Florida isolate, representing an unexpected level of conservation between viruses from different countries that are separated by more than 50 years in their isolation dates (in addition to the extensive laboratory passage history of TRVL-11573).

In summary, we detected a number of significant differences in both the coding and non-coding sequence of the TCRV strain TRVL-11573 sequence compared to the early sequences that have until now represented the only publically available reference for this important prototype arenavirus. It is anticipated that the availability of the first complete sequence of TCRV, covering both the coding and non-coding regions and based on modern sequencing methods, will be instrumental for future molecular and evolutionary studies of this virus.

## References

[1] Buchmeier MJ, de la Torre J, Peters CJ (2007). “Arenaviridae: the viruses and their replication” in Fields virology.

[2] Lopez N, Franze-Fernandez MT (2007). A single stem-loop structure in Tacaribe arenavirus intergenic region is essential for transcription termination but is not required for a correct initiation of transcription and replication. Virus Res.

[3] Charrel RN, de Lamballerie X, Emonet S (2008). Phylogeny of the genus Arenavirus. Curr Opin Microbiol.

[4] Garcin D, Kolakofsky D (1990). A novel mechanism for the initiation of Tacaribe arenavirus genome replication. J Virol.

[5] Perez M, de la Torre JC (2003). Characterization of the genomic promoter of the prototypic arenavirus lymphocytic choriomeningitis virus. J Virol.

[6] Pyle JD, Whelan SPJ (2019). RNA ligands activate the Machupo virus polymerase and guide promoter usage. Proc Natl Acad Sci USA.

[7] Charrel RN, de Lamballerie X (2010). Zoonotic aspects of arenavirus infections. Vet Microbiol.

[8] King AMQ, Lefkowitz E, Adams MJ, Carstens EB (2011). Virus taxonomy : ninth report of the international committee on taxonomy of viruses.

[9] Downs WG, Anderson CR, Spence L, Aitken TH, Greenhall AH (1963). Tacaribe virus, a new agent isolated from Artibeus bats and mosquitoes in Trinidad, West Indies. Am J Trop Med Hyg.

[10] Sayler KA, Barbet AF, Chamberlain C, Clapp WL, Alleman R, Loeb JC, Lednicky JA (2014). Isolation of Tacaribe virus, a Caribbean arenavirus, from host-seeking Amblyomma americanum ticks in Florida. PLoS One.

[11] Franze-Fernandez MT, Zetina C, Iapalucci S, Lucero MA, Bouissou C, Lopez R, Rey O, Daheli M, Cohen GN, Zakin MM (1987). Molecular structure and early events in the replication of Tacaribe arenavirus S RNA. Virus Res.

[12] Iapalucci S, Lopez N, Rey O, Zakin MM, Cohen GN, Franze-Fernandez MT (1989). The 5’ region of Tacaribe virus L RNA encodes a protein with a potential metal binding domain. Virology.

[13] Iapalucci S, Lopez R, Rey O, Lopez N, Franze-Fernandez MT, Cohen GN, Lucero M, Ochoa A, Zakin MM (1989). Tacaribe virus L gene encodes a protein of 2210 amino acid residues. Virology.

[14] Iapalucci S, Lopez N, Franze-Fernandez MT (1991). The 3’ end termini of the Tacaribe arenavirus subgenomic RNAs. Virology.

[15] Harmon B, Kozina C, Maar D, Carpenter TS, Branda CS, Negrete OA, Carson BD (2013). Identification of critical amino acids within the nucleoprotein of Tacaribe virus important for anti-interferon activity. J Biol Chem.

[16] Sommerstein R, Flatz L, Remy MM, Malinge P, Magistrelli G, Fischer N, Sahin M, Bergthaler A, Igonet S, Ter Meulen J, Rigo D, Meda P, Rabah N, Coutard B, Bowden TA, Lambert PH, Siegrist CA, Pinschewer DD (2015). Arenavirus glycan shield promotes neutralizing antibody evasion and protracted infection. PLoS Pathog.

[17] Murphy FA, Webb PA, Johnson KM, Whitfield SG, Chappell WA (1970). Arenoviruses in Vero cells: ultrastructural studies. J Virol.

[18] Li Z, Yu M, Zhang H, Wang HY, Wang LF (2005). Improved rapid amplification of cDNA ends (RACE) for mapping both the 5’ and 3’ terminal sequences of paramyxovirus genomes. J Virolog Methods.

[19] Tillett D, Burns BP, Neilan BA (2000). Optimized rapid amplification of cDNA ends (RACE) for mapping bacterial mRNA transcripts. BioTechniques.

[20] Troutt AB, McHeyzer-Williams MG, Pulendran B, Nossal GJ (1992). Ligation-anchored PCR: a simple amplification technique with single-sided specificity. Proc Natl Acad Sci USA.

[21] Zuker M (2003). Mfold web server for nucleic acid folding and hybridization prediction. Nucleic Acids Res.

[22] Albarino CG, Bergeron E, Erickson BR, Khristova ML, Rollin PE, Nichol ST (2009). Efficient reverse genetics generation of infectious junin viruses differing in glycoprotein processing. J Virol.

[23] Emonet SF, Seregin AV, Yun NE, Poussard AL, Walker AG, de la Torre JC, Paessler S (2011). Rescue from cloned cDNAs and in vivo characterization of recombinant pathogenic Romero and live-attenuated Candid #1 strains of Junin virus, the causative agent of Argentine hemorrhagic fever disease. J Virol.

[24] Patterson M, Seregin A, Huang C, Kolokoltsova O, Smith J, Miller M, Smith J, Yun N, Poussard A, Grant A, Tigabu B, Walker A, Paessler S (2014). Rescue of a recombinant Machupo virus from cloned cDNAs and in vivo characterization in interferon (alphabeta/gamma) receptor double knockout mice. J Virol.

[25] Martinez-Sobrido L, Giannakas P, Cubitt B, Garcia-Sastre A, de la Torre JC (2007). Differential inhibition of type I interferon induction by arenavirus nucleoproteins. J Virol.

